# Care seeking behaviour and aspects of quality of care by caregivers for children under five with and without pneumonia in Ibadan, Nigeria

**DOI:** 10.7189/jogh.08.020805

**Published:** 2018-12

**Authors:** Amir Kirolos, Adejumoke I Ayede, Linda J Williams, Kayode R Fowobaje, Harish Nair, Ayobami A Bakare, Oladapo B Oyewole, Shamim A Qazi, Harry Campbell, Adegoke G Falade

**Affiliations:** 1 Centre for Global Health Research, Usher Institute of Population Health Sciences and Informatics, University of Edinburgh, Edinburgh, Scotland, UK; 2 Department of Paediatrics, College of Medicine, University of Ibadan, Ibadan, Nigeria; 3 University College Hospital, Ibadan, Nigeria; 4 Department of Maternal, Newborn, Child and Adolescent Health, World Health Organization, Geneva, Switzerland; * Joint first authorship; † Joint senior authorship

## Abstract

**Background:**

This study aimed to investigate the differences in reported care seeking behaviour and treatment between children with pneumonia and children without pneumonia with cough and/or difficult breathing.

**Methods:**

Three hundred and two children aged 0-59 months with fast breathing pneumonia were matched with 302 children seeking care for cough and/or difficult breathing at four outpatient clinics in Ibadan, Nigeria. After follow up at home, Demographic and Health Survey (DHS) and Multiple Indicator Cluster Survey (MICS) questionnaires were administered in the community by trained field workers to gather information around care seeking delay, patterns of care seeking, appropriateness of care seeking and treatment provided once care was sought. Multivariable analysis was carried out to determine significant factors associated with care seeking delay.

**Results:**

Children with pneumonia had a significantly longer delay (median = 3d) before seeking care than those without pneumonia (median = 2d; *P* = 0.001). The length of the delay was 21% (95% confidence interval (CI) = 1%-42%) greater in those aged 0-1 month and 11% (95% CI = 5%-42%) greater in those aged 2-11 months compared to those aged 12-59 months. The length of delay was 17% (95% CI = 5%-30%) greater in rural locations than urban ones, and 33% (95% CI = 7%-51%) shorter in fathers with only primary education compared to higher education, adjusted for covariates. The range of places where care was sought showed the same distribution in those with and without pneumonia. Twenty two per cent of those with pneumonia sought care first from inappropriate providers. The number of children for whom caregivers reported having received antibiotic treatment was 92% for those with pneumonia and 84% for those without pneumonia.

**Conclusions:**

Given that children with pneumonia and cough/cold had similar patterns of reported care seeking information gathered on care seeking (type of provider visited) from DHS and MICS surveys on those with ‘symptoms of acute respiratory infection’ in this setting provide a reasonably valid indication of care seeking behaviours in children with pneumonia. There are high levels of antibiotic overuse for children with cough/cold in this setting which risks worsening antibiotic resistance.

Childhood pneumonia is a leading cause of mortality in children under five having accounted for 16% of child deaths globally in 2016 [1]. The majority of these deaths are in low and lower-middle income countries [2]. With 120 million episodes of childhood pneumonia annually and nearly 12 million episodes of acute lower respiratory tract infection resulting in hospital admissions, this represents a significant burden of disease [3,4].

Improving the care pathway for children with pneumonia is a key component of the wider strategy for reducing pneumonia mortality. Delays in seeking medical care from an appropriate provider for cases of childhood pneumonia are associated with a worse outcome [5]. Improving prompt care seeking at appropriate health care facilities for childhood pneumonia and other childhood diseases has therefore been an integral part of the integrated management of childhood illnesses strategy [6].

Effective care pathways for a child with pneumonia requires a caregiver to make the initial step of recognizing their child is unwell, make the decision to seek care and have the capacity and means to acquire appropriate medical care. A systematic review found recognition of pneumonia by caregivers in low income countries to be poor [7]. Caregivers’ poor knowledge of the symptoms and danger signs of childhood pneumonia can also contribute to delays or failure to seek care [8,9]. Other factors such as distance to health care facilities and economic status can affect caregivers’ ability to seek care. For example, a retrospective study found social beliefs and economic factors frequently contributed to delays in care seeking for fatal neonatal disease [10].

Care may be sought for childhood pneumonia from a wide variety of providers including pharmacies, private care providers, government institutions, community health workers and more informal providers such as traditional healers [7]. However, it is important for children with pneumonia that care is sought from a health provider who can accurately diagnose and initiate prompt antibiotic treatment appropriately where pneumonia is suspected. By understanding the factors behind why caregivers delay seeking care or seek care from inappropriate providers for children with pneumonia we can design interventions to improve prompt care seeking. This study therefore aimed to assess the sociodemographic characteristics associated with care seeking delay as well as to characterize where care was sought and what treatment was provided.

Care seeking data for children with symptoms of acute respiratory infection in low and lower-middle income countries are also frequently derived from Demographic and Health Surveys (DHS) and Multiple Indicator Surveys (MICS) [11,12]. However, previous evidence suggests that these surveys do not discriminate well between cases of cough/cold with difficult breathing and pneumonia [13]. This study therefore also aimed to investigate the differences in reported care seeking behaviour and treatment for children with and without pneumonia using these survey tools. This could establish whether these surveys which report mostly on children with cough/cold with difficult breathing also accurately reflect the care seeking behaviour of those with pneumonia.

## METHODS

### Study sites

This study was undertaken as part of a larger prospective observational study to validate pneumonia diagnosis by caregivers, which was conducted over a period of 19 months (12 August 2015 – 14 March 2017). The study sites were the children’s Out-Patients Clinics (OPC) of four hospitals in Ibadan: two public government secondary health facilities (Oni Memorial Children’s Hospital and Adeoyo Maternity Hospital), one private hospital (Our Lady of Apostle Catholic Hospital, Oluyoro, Oke- Ofa) and a tertiary hospital (University College Hospital). Ibadan is the capital of Oyo state in South-West Nigeria. Despite there being several other private hospitals and primary health care centres in the Ibadan metropolis area and adjacent districts, it is estimated that the majority of paediatric patients attend these four hospitals [14]. The most recent estimated population of Ibadan is 3 565 108, and children below the age of five years constitute about 25% of the population [15]. Ibadan is the third most populous city in Nigeria and was chosen as the study site to provide results for an urban African city which could be relevant for other similar settings throughout Nigeria and Africa.

### Study population

Children aged 0 to 59 months who present to the out-patient clinics of the above hospitals suffering from cough and/or difficulty breathing were selected for the study.

### Study design

As part of this larger prospective observational study children were first registered at the OPC medical records section. All children presenting with respiratory symptoms aged 0-59 months were examined and prescribed treatment by regular OPC physicians. Children were then referred to the study physicians where diagnosis of those with or without pneumonia was confirmed by the trained study physicians using standard WHO pneumonia management protocols [16]. Baseline forms which included all their personal details for the recruited children were filled out.

Individual interviews were subsequently held with mothers/caregivers of these children followed up at home two or eight weeks following attendance at OPCs using Demographic and Health Survey (DHS5) and Multiple Indicator Survey (MICS5) questionnaires. This was done as per the study protocol of the larger prospective observational study which randomly assigned matched cases to either two or eight week follow up [17]. Doctors, who had completed training on how to carry out these demographic surveys, interviewed the caregiver of each child in their home using DHS5 and MICS5 questionnaires to elicit care seeking information for the episode of respiratory illness.

A WHO staff member with substantial experience in training for pneumonia diagnosis and management, Dr S.A. Qazi, visited the study sites on two occasions: first, before enrolment started and second, toward the middle of the study period on May 29 to June 3, 2016 to monitor the conduct of and progress with the study. This provided high quality training in pneumonia diagnosis using WHO criteria as well as supervision for study physicians.

### Selection criteria

Selection criteria was based on the study protocol for the wider observational study [17].

Inclusion criteria:

1. Children aged 0 to 59 months who present to the out-patient departments of the study hospitals suffering from cough and/or difficult breathing and
2. Caregiver willing to sign informed consent form.

Exclusion criteria:

1. Children presenting primarily with an episode of recurrent wheeze or asthma
2. Children who had pneumonia with presence of danger signs, severe enough to require hospital admission
3. Children who had symptoms of more than four weeks’ duration
4. Children who had previously come to the same health facility for treatment of the same illness episode and had already been enrolled in the study
5. Children having history of recent pneumonia episode within the past 10 days
6. Children with history of congenital heart disease (suspected or confirmed)
7. Children who lived outside Ibadan (to facilitate appropriate follow-up)
8. Children whose legal guardian did not give consent

### Data collection

The sequence of recruitment of these children in the outpatient departments was as follows:

- Children were first registered at the OPC medical record section. All children aged 0-59 months were then referred to OPC.
- The regular OPC physicians examined and prescribed treatment to all children with cough and/or difficulty breathing. These children were then referred to the research study physicians.
- The study physician then re-evaluated these children according to the WHO case management protocol [16] and confirmed whether the child actually had pneumonia or not. They then confirmed children matched the selection criteria.
- An informed written consent to participate was then taken from the caregivers.
- The study physician then proceeded with the enrollment. Baseline forms which included all their personal details were filled out for the recruited children.
- The study physician then recorded the key clinical and treatment details.

### Data management

Double data entry and cleaning was undertaken using EpiData software (EpiData Association, Odense, Denmark). Descriptive statistics were used to assess the socio-demographic and clinical characteristics of the study children.

Pneumonia cases were matched with “no pneumonia” cases at the end of the study on the basis of sex, age, study physician who assessed them and week of follow up. The matching procedure is described in more detail in the study protocol of the wider observational study [17].

### Data extraction and statistical analyses

Data analyses were carried out on the data extracted on matched cases of pneumonia and “no pneumonia” in children aged 0-59 months. Questions on socio-demographic characteristics of the child/caregiver, how long it took the caregivers to seek care (delay) for the sick child, type of treatment provided, number of medications prescribed and where care was first sought were extracted from the pooled data set using the DHS5 & MICS5 questionnaires (Appendix S1 in **Online Supplementary Document(Online Supplementary Document)**). All data were re-coded during data cleaning with no data linked to individuals.

Appropriate and inappropriate care provider definitions within DHS and MICS surveys vary between countries. For this study, we defined appropriate providers as those in government or private hospitals and clinics. Other care sources including pharmacies, chemists, self-bought medicines, friends/relatives, Non-governmental organizations (NGOs), other private medical facilities which were not clinics nor hospitals and other non-specified sources were defined as inappropriate care providers.

Descriptive statistical analyses of the location where care was sought, and the type of care received at inappropriate providers were performed. Data analyses and management were carried out using STATA version 13. Chi-square (χ^2^) analysis was used to examine the association between the socio-demographic characteristics of the child/caregiver with where treatment was, sought as well as the time taken (delay) to seek care for the sick child with pneumonia. A *P*-value of <0.05 was taken as significant. Significant variables were then included in a poisson regression model with multiple covariates to identify significant factors associated with care seeking delay. These analysed differences as a rate ratio and care seeking delay was analysed as a continuous variable.

### Ethics statement

Ethical approval for the study was obtained from the University of Ibadan / University College Hospital Ibadan Institutional Review Board, Oyo State Ministry of Health Ethical Committee and the WHO Ethics Review Committee. Further permission was obtained from the authorities of each participating hospital prior to the conduct of the study and informed consent was obtained from the caregivers of the study children.

## RESULTS

### Socio-demographic characteristics of study population

A total of 302 confirmed pneumonia cases were included; 168 males and 137 females, aged 0-52 months (Mean = 13.8 months, SD = 12.1). A total of 360 children without pneumonia were recruited into the study. This recruitment is detailed in a separate study [17]. 302 children of those without pneumonia were included as a control group having been matched to cases of pneumonia; 168 males and 137 females, age 0-52 months (Mean = 15.9, SD = 13.7). The socio-demographic characteristics of those in the study are detailed in **Table 1**.

**Table 1:** Socio-demographic characteristics of study population

Characteristics	Pneumonia n = 302 (%)	No pneumonia n = 302 (%)
**Age of child (months) (mean ± SD)**	13.8 ± 12.1	15.9 ± 13.7
**Age category of child:**		
0 to 1 months	22 (7.3)	33 (10.9)
2 to 11 months	141 (46.7)	123 (40.7)
12 to 59 months	139 (46)	146 (48.3)
**Gender:**		
Male	168 (55.6)	168 (55.6)
Female	134 (44.4)	134 (44.4)
**Siblings:**		
No siblings	95 (31.5)	109 (36.1)
One or more	207 (68.5)	193 (63.9)
**Age category of mothers:**		
≤30 years	119 (39.4)	130 (43)
>30 years	183 (60.6)	172 (57)
**Mother’s education:**		
None/primary	15 (5)	20 (6.6)
Secondary	110 (36.4)	96 (31.8)
More than secondary	177 (58.6)	186 (61.6)
**Father’s education:**		
None/primary	9 (3)	8 (2.6)
Secondary	88 (29.1)	103 (34.1)
More than secondary	205 (67.9)	191 (63.3)
**Father’s employment status:**		
Unemployed	7 (2.3)	7 (2.3)
Employed	295 (97.7)	295 (97.7)
**Location:**		
Rural	56 (18.5)	62 (20.5)
Urban	246 (81.5)	240 (79.5)

### Delay in care seeking

**Figure 1:**
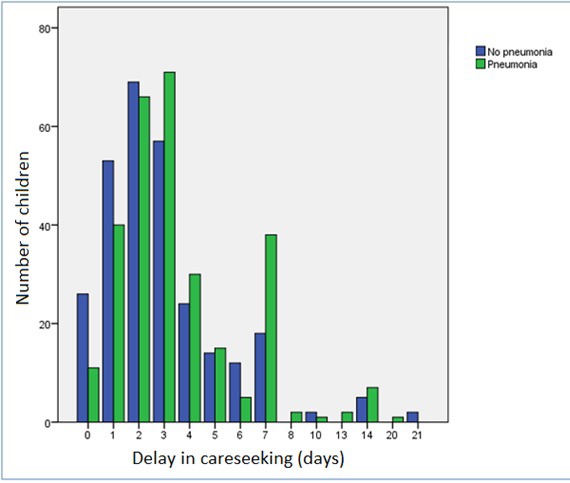
Care seeking delay in days by diagnosis.

The mean delay before seeking care for the pneumonia group was 3.6 days (median 3.0, interquartile range IQR = 2-4, range = 0-20). For the group without pneumonia the mean delay was 3 days (median = 2.0, IQR = 1-4, range = 0-21). Differences between the two groups were studied by means of a Mann-Whitney U test and revealed a statistically significant difference in median delay (*P* = 0.001). **Figure 1** displays the reported delays in seeking care for both the pneumonia and “no pneumonia” groups.

Mother’s age, number of siblings and child’s gender were all found to be non-significant variables affecting care seeking delay when assessed in a univariable analysis. These variables were therefore not included in the multivariable analysis. **Table 2** shows the results of a multivariable effects model which assessed a number of demographic characteristics (child’s age, mother’s education, father’s education, urban/rural resident status) in relation to care seeking delay. The multivariable model found significant differences in care seeking based on child’s age, rural/urban resident status and paternal education. However, despite being significant in the univariable model, maternal education was no longer statistically significant when analysed in the multivariable model. The length of the care seeking delay was 21% greater in the 0-1 month group, and 11% greater in the 2-11 months group compared to the 12-59 months group. Similarly, the length of care seeking delay was 17% greater for those in rural locations than those in urban ones. The length of delay was 19% greater in mothers with only at most primary education however this was not statistically significant (*P* = 0.077). The care seeking delay for children whose fathers had up to primary education was 33% lower than those fathers who had higher education.

**Table 2:** Multivariable effects model of factors influencing care seeking delay

Demographic characteristic	Relative rate of care seeking delay	95% CI	*P*-value
Age 12-59 months (**Ref.**)	–		
Age 2-11 months	1.11	(1.05-1.42)	0.019
Age 0-1 months	1.21	(1.01-1.22)	0.038
Urban location (**Ref.**)	–		
Rural location	1.17	(1.05-1.3)	0.006
Secondary maternal education (**Ref.**)	–		
None/primary maternal education	1.19	(0.98-1.45)	0.077
Secondary paternal education (**Ref.**)	–		
None/primary paternal education	0.67	(0.49-0.93)	0.015

SD – standard deviation, CI – confidence interval

Inclusion in the multivariable model of pneumonia status allowed assessment of difference in care-seeking delay between those with and without pneumonia adjusted for sociodemographic characteristics. The difference in care-seeking delay remained statistically significant with care seeking delay 20% greater in those with pneumonia compared to those without (relative rate of care seeking delay = 1.20, 95% CI = 1.09-1.31).

### Appropriateness of care seeking

**Table 3** shows where care was first sought for children with and without pneumonia. Government hospitals were the most common place to first seek care for both groups. Of those who had pneumonia, 78% first sought care from appropriate care providers which we defined as government hospitals, health centres, health posts and private hospitals or clinics. The pattern of where care was sought was largely similar between those with and without pneumonia. **Table 4** shows the delay in care seeking for both groups seeking care initially from appropriate and inappropriate providers. Significance testing (Mann-Whitney U test) suggested no evidence that delay in seeking care was related to appropriateness of consultation source for both those with pneumonia (*P* = 0.08) and without pneumonia (*P* = 0.5).

**Table 3:** Source of first consultation by diagnosis

Source of first consultation	No pneumonia n = 302	Pneumonia n = 302	Total
Government hospital*	183	171	354
Government health centre*	14	17	31
Government health post*	1	0	1
Private hospital/clinic*	39	48	87
Pharmacy/chemist/patent Medicine	59	59	119
Other private medical	2	2	4
Friend/relation	4	2	6
NGO	0	1	1
Other	0	2	2

NGO – non-governmental organisation

*Considered appropriate health care providers.

**Table 4:** Delay in care seeking for those seeking care initially from appropriate and inappropriate providers

		Number seeking care	Number of cases with missing care seeking delay data*	Median delay in days (IQR), n
**Appropriate health care provider†**	No pneumonia	237	18 (8%)	2 (1-3.5), n = 219
	Pneumonia	236	10 (4%)	3 (2-5), n = 226
**Inappropriate health care provider‡**	No pneumonia	65	2 (3%)	3 (1-4), n = 63
	Pneumonia	66	3 (5%)	3 (1.5-4), n = 63

IQR – interquartile range

*Represents the number of cases with incompletely recorded data on care seeking delay within each sub category.

†Government hospitals, health centres, health posts and private hospitals and clinics were considered appropriate providers.

‡Pharmacies, chemists, friends, relations, other private facilities, and other sources were considered inappropriate providers.

### Care provision

Of children taken to inappropriate care providers, which mostly consisted of those attending pharmacists/chemists, 63% (36/57) of children with pneumonia and 68% (40/59) of children without pneumonia were given advice on what medication to buy. Of children taken to inappropriate care providers, only 12% (7/58) of children with pneumonia were examined and no children without pneumonia were examined.

**Table 5** provides details of the management provided to those seeking care by pneumonia status. Eight per cent (23/300) of cases with pneumonia aged 12-59 months had a chest x-ray ordered as part of their investigations. Ninety two per cent (278/301) of children with pneumonia were given antibiotics, however 84% (253/302) of those without pneumonia also received antibiotics. Antibiotics given to those with and without pneumonia mainly consisted of penicillins and cephalosporins. Other medicines apart from antibiotics were also given to the majority of both pneumonia and “no pneumonia” cases (Appendix S2 in **Online Supplementary Document(Online Supplementary Document)**). Seventy two percent (217/302) of those with pneumonia and 71% (215/302) of those without pneumonia received four or more drugs.

**Table 5:** Type of treatment provided or investigation requested by diagnosis

	No pneumonia (n = 302)	Pneumonia (n = 302)*
**Chest radiography ordered:**		
Yes	5 (2%)	23 (8%)
No	297 (98%)	277 (92%)
**Antibiotics:**		
Yes	253 (84%)	278 (92%)
No	49 (16%)	23 (8%)
**Antimalarial:**		
Yes	97 (32%)	94 (31%)
No	205 (68%)	207 (69%)
**Other medicines:**		
Yes	276 (91%)	263 (87%)
No	26 (9%)	38 (13%)
**Number of drugs:**		
1	9 (3%)	8 (3%)
2	16 (5%)	25 (8%)
3	62 (21%)	52 (17%)
4+	215 (71%)	217 (72%)

*Data on treatment provided was incomplete for two cases of pneumonia.

## DISCUSSION

This study compared the care seeking behaviours for caregivers of children with and without pneumonia who presented to four study sites in Ibadan, Nigeria. The patterns of where care was sought were broadly the same between those with and without pneumonia. Twenty-two per cent of those with pneumonia sought care first from inappropriate health care providers where very few children were examined for signs of pneumonia. The high number of children without pneumonia given antibiotics indicates a high level of inappropriate antibiotic prescribing in this setting. This study also found children with pneumonia had a longer mean delay of 0.6 days before seeking care than those without pneumonia. The length of delay was greater in young children and those resident in rural areas. Surprisingly, care seeking delay was also greater for those children with fathers who had higher education compared to those with up to primary education.

The patterns of where health care was sought were broadly similar in those with and without pneumonia in this study. However, there were significant differences between those with and without pneumonia in terms of the length of delay. Reported care seeking behaviours for groups of children with cough/cold with difficult breathing are therefore likely to reflect the care seeking patterns for children with pneumonia in terms of where care was sought, but not in terms of delay. This finding may also indicate that the place where care is sought may be more related to access rather than caregiver characteristics, presenting complaints or severity of symptoms. In addition, this finding may have been influenced by restrictions in the range of options where care can be sought in the area.

Twenty-two per cent of children with pneumonia sought care first from inappropriate health care providers, which were defined as pharmacies/chemists, friends/relatives and other facilities out with government health facilities or private hospitals/clinics. Diagnosing pneumonia effectively and accurately is important so that appropriate treatment with antibiotics can be provided. However, the very low number of children examined when seeking care from inappropriate health care providers illustrates that these places are unlikely to provide adequate care for children with pneumonia and that the current classification of these providers as ‘inappropriate’ is valid with respect to child pneumonia.

This study found that the majority of children with pneumonia were given antibiotics. However, it was also found that a very high proportion of those without pneumonia were also given antibiotics. This represents a high level of inappropriate prescribing for those with cough/cold. This finding is of significant concern, as high rates of antibiotic prescribing for those with cough/cold is likely to drive antimicrobial resistance and to increase health care costs unnecessarily. The increasing problem of antimicrobial resistance is a major problem within Nigeria and across Africa [18,19]. This study is in a location which is similar to other urban sites in Nigeria and throughout Africa. Inappropriate prescriptions of antibiotics have been a persistent problem in low income settings [20]. There has also been a push to reduce overprescription of antimicrobials and promote their rational use with standard case management [21]. Efforts to promote the judicious use of antibiotics for children with respiratory illnesses while maintaining high rates of appropriate treatment with antibiotics for children with pneumonia should therefore continue. These aspects which reflect inappropriate care require action through appropriate training, supervision and mentorship of those working in clinical settings as well as national strategies for education and training in appropriate practice.

DHS and MICS questions enquire about fast, short, shallow breathing and respiratory distress in the previous two weeks (and whether these were chest-related) [11,12]. DHS and MICS are not designed to specifically identify cases of pneumonia and they label these episodes as ‘symptoms of acute respiratory infection (ARI)’. One prospective study in Pakistan and Bangladesh also found current methods discriminate poorly between children with pneumonia and those with cough/cold resulting in a poor yield of those who have pneumonia [13]. Therefore, a large proportion of the data gathered for care seeking from these surveys actually reflect the behaviours of those seeking care for children with symptoms of ARI (mostly comprising children with cough/cold). Nevertheless, our data suggest that reported care seeking behaviours of children with symptoms of ARI in surveys such as DHS and MICS will accurately reflect where care was sought for children with pneumonia. They may be less valid however in representing the length of care seeking delay for children with pneumonia and this finding may require further investigation.

The reasons behind the difference in delay in care seeking found in this study, with a longer delay for those with pneumonia compared to those with cough/cold, are not clear. This is an unexpected finding given that pneumonia cases would likely develop more severe symptoms. This result was statistically significant, however we acknowledge that a mean difference of 0.6 days in this study may not necessarily reflect a clinically significant difference between the two groups. We suggest that further investigation may be needed in other contexts to see if this result is reproducible. Higher paternal education was associated with a longer care seeking delay and this is also a surprising finding. This may perhaps be due to greater confidence in the ability to manage childhood respiratory illnesses at home or due to other differing factors in those with further education. Other studies have found maternal education to influence care seeking decisions [22]. Higher maternal education was associated with shorter care seeking delay, however this difference was not statistically significant in the multivariable effects model in this study. This may indicate that care seeking decisions for children with pneumonia depend more on paternal decision making. Rural status was associated with longer care seeking delay, and this has previously been found in other studies [22-24]. Longer delay was found in younger children compared to those in older age categories. Different signs and symptoms of pneumonia in different age groups may be a factor in these differences for care seeking delay or may also be due to superstition or traditional beliefs. Care seeking delays were notably not significantly different in girls and boys. Previous analyses of DHS data on care seeking based on gender have revealed a complex relationship for differences in care seeking between genders and that these differences vary between different contexts and over time [25].

### Strengths and limitations

This study was conducted as part of a larger prospective study investigating caregiver recall of childhood pneumonia in outpatient facilities from one city in Nigeria. This may limit how generalisable these results are in other areas and further investigation in consistency of findings across settings would be of use. This sample may also not entirely represent those normally surveyed by DHS and MICS in the community as they may have different characteristics. There was also some loss to follow up in those recruited and therefore this may have introduced some bias [17]. OPC physicians were aware of the study and referred children to the study physicians. This knowledge may have influenced their regular practice in terms of care provided for a number of children. The completeness of data recording was high and only a few questionnaires had incomplete data, which did not bias the results substantially. Some antibiotic use in cough/cold cases may have been justified due to a concurrent illness requiring antibiotics, but the high use reported in this study was definitely inappropriate. Care seeking data based on caregiver report from surveys can have potential recall bias or information error. There is some “heaping” of reported care seeking delay at days 7 and 14 (**Figure 1**) which likely represent a preference for reporting in weeks as opposed to the accurate number of days for a number of cases. This recall bias should be acknowledged and accounted for in the analysis of larger DHS and MICS survey data which enquire about care seeking delay. There is the potential to use statistical methods to account for heaping in retrospectively reported data where this may influence results [26].

## CONCLUSIONS

We found a high rate of antibiotics being prescribed correctly for children with pneumonia but also to those presenting with cough/cold, which contributes to an increase in antimicrobial resistance. The high rate of antibiotic treatment for children with pneumonia should be maintained however; training and education for care providers on diagnosis and appropriate prescribing with antibiotics is required. This should continue alongside supervision and monitoring of practices as part of the strategy to reduce the burden of antimicrobial resistance. Factors including rural location and the age of children influence delays in care seeking and interventions should be identified to address these key areas. The influence of these factors and others, including gender and maternal education, likely vary between countries and contexts. Continued investigation in different settings will help characterise further how these factors influence care seeking. Data from this study suggests that information gathered on several aspects of care seeking from DHS and MICS surveys on children with symptoms of ARI may provide a reasonable indication of care seeking patterns for children with pneumonia. The study supports DHS and MICS classification of “inappropriate providers” since it was reported that they rarely examine children and so cannot identify children with pneumonia and give appropriate care. Care seeking for nearly a quarter of children with pneumonia occurs initially at these inappropriate care providers which is of serious concern.
