# Supplementary material for: Care seeking behaviour and aspects of quality of care by caregivers for children under five with and without pneumonia in Ibadan, Nigeria
Source: J Glob Health. 2018 Sep 19;8(2):020805. doi: 10.7189/jogh.08.020805 (PMC6150609; doi:10.7189/jogh.08.020805)
Supplement: Online Supplementary Document [file jogh-08-020805-s001.pdf]

Online Supplementary Document

Kirolos et al. Care seeking behaviour and aspects of quality of care by caregivers for children under five with and without pneumonia in Ibadan, Nigeria

J Glob Health 2018;8:020805

Appendix S1: DHS5 and MICS5 questionnaires used

DHS QUESTIONNAIRE

533 *Has (NAME) been ill with a fever at any time in the last 2 weeks?*

Yes	*1*
No	*2*
Don't Know	*8*

534 *Has (NAME) had an illness with a cough at any time in the last 2 weeks?*

Yes	*1*
No	*2*
Don't Know	*8*

535 *When (NAME) had an illness with a cough, did he/she breathe faster than usual with short, rapid breaths or have difficulty breathing?*

Yes	*1*
No	*2*
Don't Know	*8*

536 Was the fast or difficult breathing due to a problem in the chest or to a blocked or runny nose?

Chest Only	*1*
Nose Only	*2*
Both	*3*
Other _______________ (Specify)	*4*
Don't Know	*8*

537 Check 533: Had Fever?

538 Now I would like to know how much (NAME) was given to drink (including breastmilk) during the illness with a (fever/cough). Was he/she given less than usual to drink, about the same amount, more than usual to drink?
If less, probe: Was he/she given much less than usual to drink or somewhat less?

Much Less	*1*
Somewhat Less	*2*
About the Same	*3*
More	*4*
Nothing to Drink	*5*
Don't Know	*8*

539 *When (NAME) had a (fever/cough), was he/she given less than usual to eat, about the same amount, more than usual, or nothing to eat?*
If less, probe: Was he/she given much less than usual to eat or somewhat less?

Much Less	*1*
Somewhat Less	*2*
About the Same	*3*
More	*4*
Stopped Food	*5*
Never Gave Food	*6*
Don't Know	*8*

540 *Did you seek advice or treatment for the illness from any source?*

Yes	*1*
No	*2*

541 *Where did you seek advice or treatment? Anywhere else?*
Probe to identify each type of source and circle the appropriate code(s).

If unable to determine if a hospital, health center or clinic is public or private medical, write the name of the place.

____
(Name of place)

Public Sector

Govt Hospital	*A*
Govt Health Center	*B*
Govt Health Post	*C*

Mobile Clinic	*D*
Fieldworker	*E*
Other Public	*F*
________________ *(specify)*	

Private Medical Sector

PVT Hospital/Clinic	*G*
Pharmacy	*H*
PVT Doctor	*I*
Mobile Clinic	*J*
Fieldworker	*K*
Other Private Med.	*L*
________________ *(specify)*	

Other Source

Shop	*M*
Traditional Practitioner	*N*
Other	*X*
________________ *(specify)*	

543 Where did you first seek treatment? Use letter code from 541.

544 How many days after the illness began did you first seek advice or treatment for (NAME)? If the same day, record '00'.

545 Is (NAME) still sick with (fever/cough)?

Fever Only	*1*
Cough Only	*2*
Both Fever and Cough	*3*
No, Neither	*4*
Don't Know	*8*

546 At any time during the illness, did (NAME) take any drugs for the illness?

Yes	*1*
No	*2*

547 What drugs did (NAME) take? Any other drugs? Record all mentioned.

Antimalarial Drugs	
SP/Fansidar	*A*
Chloroquine	*B*
Amodiaquine	*C*
Quinine	*D*
Artemeter / Lumefantrin	*E*
Atesunate / Amodiaquine	*F*
Other Antimalarial	*G*
_________________ (specify)	
Antibiotic Drugs	
Pill/Syrup	*H*
Injection	*I*

Specify Name of Antibiotic ________________

Other Drugs

Aspirin	*J*
Acetaminophen	*K*
Ibuprofen	*L*
Other ________________ (specify)	*X*
Don't Know	*Z*

Followed by specific questions about each drug mentioned…

MICS QUESTIONNAIRE

CA7. AT ANY TIME IN THE LAST TWO WEEKS, HAS (*name*) HAD AN ILLNESS WITH A COUGH?	Yes .... 1<br>No .... 2<br><br>DK 8	2⇨CA14<br><br>8⇨CA14
CA8. WHEN (*name*) HAD AN ILLNESS WITH A COUGH, DID HE/SHE BREATHE FASTER THAN USUAL WITH SHORT, RAPID BREATHS OR HAVE DIFFICULTY BREATHING?	Yes .... 1<br>No .... 2<br><br>DK 8	2⇨CA14<br><br>8⇨CA14
CA9. WAS THE FAST OR DIFFICULT BREATHING DUE TO A PROBLEM IN THE CHEST OR A BLOCKED OR RUNNY NOSE?	Problem in chest .... 1<br>Blocked or runny nose .... 2<br><br>Both .... 3<br><br>Other (*specify*)__________ 4<br>DK 8	2⇨CA14<br><br><br>6⇨CA14
CA10. DID YOU SEEK ANY ADVICE OR TREATMENT FOR THE ILLNESS FROM ANY SOURCE?	Yes .... 1<br>No .... 2<br><br>DK 8	2⇨CA12<br><br>8⇨CA12
CA11. FROM WHERE DID YOU SEEK ADVICE OR TREATMENT?		
CA12. WAS (NAME) GIVEN ANY MEDICINE TO TREAT THIS ILLNESS?	Yes .... 1<br>No .... 2<br><br>DK 8	2⇨CA14<br><br>8⇨CA14
CA13. WHAT MEDICINE WAS (NAME) GIVEN?<br><br>PROBE:<br>ANY OTHER MEDICINE?<br><br>CIRCLE ALL MEDICINES GIVEN. WRITE BRAND NAME(S) OF ALL MEDICINES MENTIONED.<br><br><br>(NAMES OF MEDICINES)	Antibiotic<br>Pill / Syrup .... A<br>Injection .... B<br><br>Anti-malarials .... M<br><br>Paracetamol / Panadol / AcetaminophenP<br>Aspirin .... Q<br>Ibuprofen .... R<br><br>Other (specify) .... X<br>DK .... Z	

Appendix 2 - Pattern of medications other than antibiotics prescribed for those with and without pneumonia

Table A1 Pattern of medications other than antibiotics prescribed for those with and without pneumonia by age group

	Age groups		
**Medication**	**0-1 months** n=22	**2-11 months** n=141	**12-59 months** n=139
**Pneumonia**			
Antimalarials*	**3**	**37**	**81**
Artemether lumefantrine	1	9	26
Artesunate amodiaquine	2	24	49
Dihydoartemisinin piperaquine	0	0	2
Quinine	0	4	0
Artesunate	0	0	1
Arthemether	0	0	1
Proguanil	0	0	2
Other drugs*			
Cough syrup	3	35	67
Analgesic/antipyretic	10	60	79
Anti-histamines	2	6	7
Anti-helminthics	0	0	4
Eye/nasal drops	2	18	5
Minerals and vitamins supplements	23	121	89
Natural health/herbal products	2	4	2
Probiotics	0	2	3
Nasal decongestants	0	2	0
Oral Rehydration solutions	0	3	1
Topical creams/ lotions	2	2	1

Salbutamol	1	5	2
	**No Pneumonia**		
**Medication**	**0-1 months** n=33	**2-11 months** n=123	**12-59 months** n=146
Antimalarials*	**2**	**39**	**75**
Artemether lumefantrine	0	7	19
Artesunate amodiaquine	2	29	51
Dihydoartemisinin piperaquine	0	1	0
Quinine	0	2	2
Arthemether	0	0	3
Other drugs*			
Cough syrup	7	58	56
Analgesic/antipyretic	7	52	79
Anti-histamines	7	9	28
Anti-helminthics	0	0	3
Eye/nasal drops	4	12	6
Minerals and vitamins supplements	27	83	94
Natural health/herbal products	2	5	0
Probiotics	0	2	6
Nasal decongestants	0	1	1
Rehydration solutions	0	9	9
Saline irrigation of the eye	0	2	1
Topical creams/ lotions	0	1	2
Salbutamol	0	2	0
Antiseptics/ disinfectants	0	1	0

*Some children got more than one drug type
